# Supplementary material for: Dendritic Cell-Based Therapeutic Immunization Induces Th1/Th17 Responses and Reduces Fungal Burden in Experimental Sporotrichosis
Source: Microorganisms. 2025 Oct 14;13(10):2351. doi: 10.3390/microorganisms13102351 (PMC12566472; doi:10.3390/microorganisms13102351)
Supplement: Supplementary file 1 [file microorganisms-13-02351-s001.zip › microorganisms-3887369-supplementary.pdf]

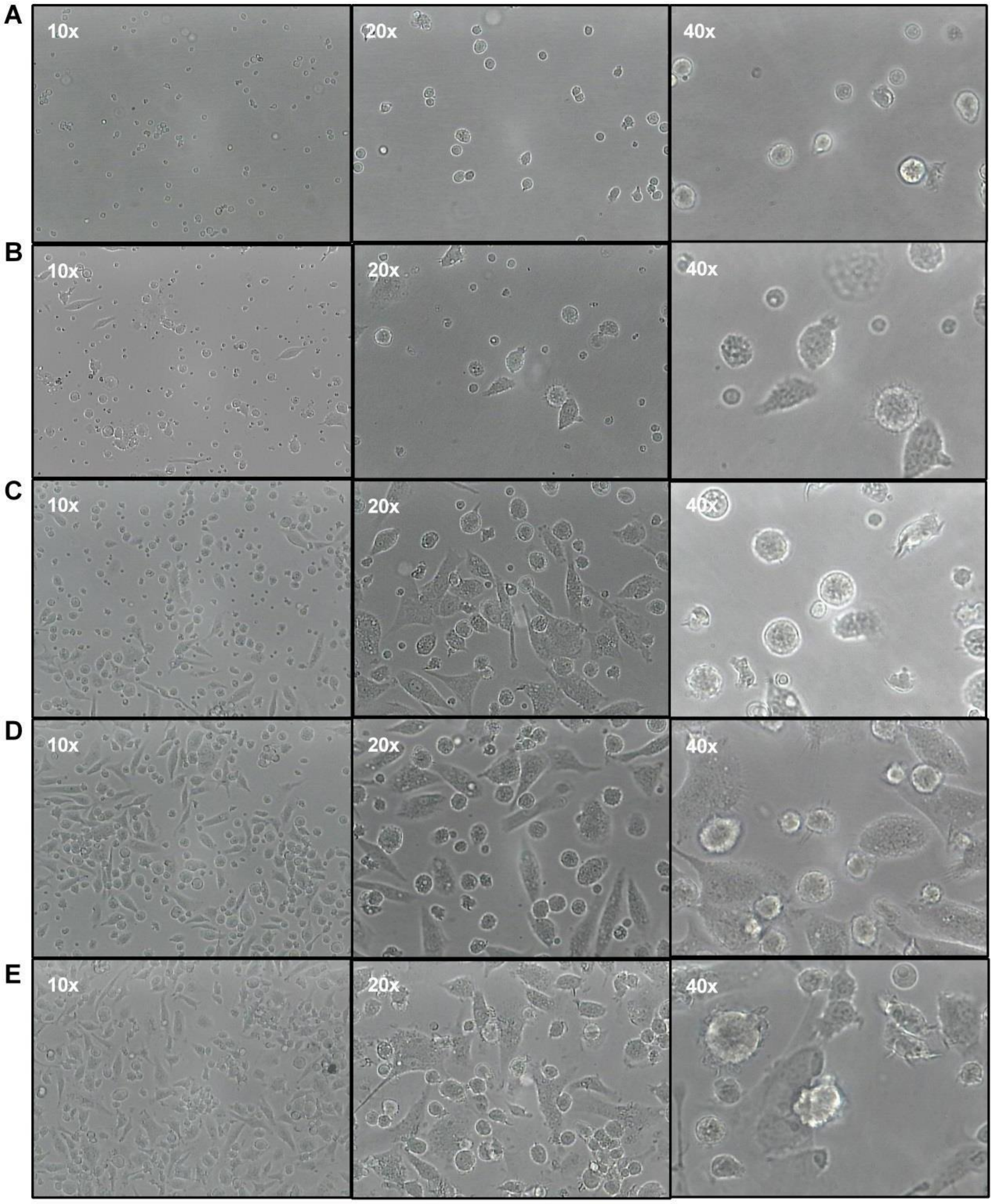

Supplementary Figure S1. Optical microscopy images showing the morphological changes during BMDC differentiation. Images were acquired using a Nikon Eclipse TS100 inverted optical microscope at 10×, 20×, and 40× magnification. Precursor cells are shown at the start of differentiation (A) and after 3 (B), 6 (C), 8 (D), and 10 (E) days of culture.

.
